# Glances at the Hospitals

**Published:** 1904-12-31

**Authors:** 


### GLANCES AT THE HOSPITALS.

### STAMFORD AND RUTLAND INFIRMARY.

THE income amounted to £2,969, and the expenditure to £2,446; but on January 1st there was an adverse balance of £457 which has been something more than cleared off during the year. At the beginning of the year there were 18 patients in residence, and 262 were admitted, making a total of 280 under treatment during the year. Of these 144 were discharged cured, 60 discharged relieved, 10 discharged incurable, 5 at own request, 29 were made out-patients, 12 died, and 20 remained on the last day of December. The mortality was 4·6 per cent. In addition to the above numbers, 20 cases were treated in the isolation department, 19 being cured and 1 remained in the wards. Of these isolation ward cases, 17 were of scarlatina and all of them recovered. Of 4 cases of rheumatic fever all were cured. Of 8 cases of appendicitis, 6 were cured, 1 relieved, and 1 died. The operation table shows that 110 operations were performed, but no results of these operations are given. We are, however, glad to note the insertion of an anæsthetic table, from which we learn that ether was administered 18 times, chloroform 66 times, A.C.E. mixture 11 times, chloroform and ether 12 times, and nitrous oxide 3 times.

### ROYAL HOSPITAL, RICHMOND, SURREY.

THE receipts during the year amounted to £7,115, but £3,391 were legacies, and were for the most part transferred to the new building fund account, so that there was a balance of £217 on the ordinary working of the institution due to the treasurer at the end of the year. The average number of in-patients was 49, and 660 were admitted. The average cost of each bed occupied was £75 17s.; the average cost of each in-patient was £5 12s. and the average cost per day was 4s. 1¾d. The medical and surgical tables merely show the numbers under treatment for the various diseases and are therefore comparatively useless. There is no operation table and no anæsthetic table, although 344 operations were performed. There were 52 deaths, and as the causes of death are given it is possible to arrive at some definite information in certain cases. For instance, one table tells us there were 12 cases of pneumonia, and by referring to the mortality table we find 3 deaths from that disease, and we guess that 9 cases were either cured or relieved; and the same process may be followed in other diseases.

### CHILDREN'S HOSPITAL, WALTHAMSTOW.

THE total ordinary income was £1,553, and the total ordinary expenditure was £1,739, leaving a deficit of £186. The committee express the hope that this will be wiped off by some friends of the hospital. The additions were opened in July 1903, by the Duchess of Connaught, patroness of the hospital, and the institution has now a thoroughly equipped out-patient department, an operating-room, a casualty-room, and a mortuary, besides other additions to the main structure which have increased the efficiency of the management and the comfort of the resident staff. But of course a larger hospital means a larger expenditure and hence it is essential that the subscription list be increased. At the opening ceremony purses were presented by 60 children and these contained £334. Then Mr. Warner gave £250 which, added to his former gift, made £1,750. On January 1st there were 13 patients in the hospital and 573 were admitted during the year, making a total of 586 under treatment. Of these 211 were medical, 350 surgical, and 25 gynæcological. Among the medical diseases we notice that there were 31 cases of pneumonia, and of these 21 were cured, 7 died, and 3 remained in the wards. Twenty-two cases of enteric fever resulted in recovery in 13, death in 6, and 3 were under treatment on the date of the report. Of 25 cases of acute rheumatism, 23 were cured and 2 were still in the hospital. An analysis of the 586 cases shows that 498 were cured, 19 improved, 8 not improved, 34 died, and 27 were in the wards. Anæsthetics were administered 291 times. Nitrous oxide was given 73 times, ether 7 times, chloroform 188 times, A.C.E. mixture 7 times, and nitrous oxide and ether 16 times.

### THE HEREFORDSHIRE GENERAL HOSPITAL.

THE ordinary receipts amounted to £5,056, and over £1,000 was received in legacies. The expenditure amounted to £3,896. It must be noted the subscriptions have increased by £422, the hospital ball produced £130, and Mr. George Cresswell proposed and carried out a scheme whereby one penny a month is collected from every house in the county. This was begun in May and during the seven months it produced £708. Here is an example for other hospitals to follow, and it has been partly instrumental in enabling the board to replace £1,000 which had been drawn from the capital account. On January 1st there were in residence 54 patients, and 634 were admitted during the year, making 688 under treatment. Of these 483 were discharged cured, 97 were discharged relieved, 12 not relieved, 35 died, and 61 remained in the wards. The medical, surgical, and operation tables are all compiled carefully, and give sufficient information. There were 342 operations, many of them being severe, and only 2 deaths. The anæsthetic table shows that chloroform was given 170 times, ether 157, gas 57, and eucaine 8. Speaking generally the report is a good one, but we miss a statement of the cost per bed occupied, the cost per patient, the cost per diem, and the average duration of treatment.

